# A Rare Cause of Right Lower Quadrant Abdominal Pain: Uncomplicated Ileal Diverticulitis

**DOI:** 10.7759/cureus.54887

**Published:** 2024-02-25

**Authors:** Alexandria L Betit, Ty K Stansell

**Affiliations:** 1 Department of Physiology, Alabama College of Osteopathic Medicine, Dothan, USA; 2 Family Medicine, Northeast Alabama Regional Medical Center, Anniston, USA

**Keywords:** case report, small bowel, rlq abdominal pain, uncomplicated ileal diverticulitis, diverticulitis, ileum

## Abstract

Ileal diverticulitis is a rare cause of abdominal pain. Even though small intestine diverticulosis is relatively rare, resulting pathologies including diverticulitis are still clinically relevant in both an inpatient and outpatient setting often presenting with varying levels of severity. Most reported cases of ileal diverticulitis are complicated and managed surgically. In contrast to these more complicated presentations, this report illustrates an uncomplicated case of ileal diverticulitis presenting with right lower quadrant abdominal pain and leukocytosis treated successfully conservatively with medical management. Although rare, uncomplicated ileal diverticulitis is clinically pertinent and should be included in the differential diagnosis of abdominal pain as this disease presentation can cause symptoms that are commonly associated with more prevalent pathologies such as acute appendicitis. Thus, these presentations are often mistaken for other more common and/or emergent pathologies depending on the region of the symptomatic small bowel diverticulitis. In this case report, the patient’s presentation initially mimicked mild atypical appendicitis and was thus managed with this diagnosis in mind without the need for more extensive treatment including surgery.

There are currently fewer case reports available that display a patient with uncomplicated ileal diverticulitis and the difference in the presentation and management of these patients compared to more severe cases. Physicians should have a heightened awareness of this disease process to avoid delayed management or prevent/postpone complications. This lack of current awareness in part may be due to the less volatile presentation associated with uncomplicated ileal diverticulitis and thus delayed patient presentation, as was seen with this case. However, it is important to note that as with any patient’s care, proper treatment must be individualized, especially given the variable nature of patient presentations with ileal diverticulitis. All in all, one hope is that improving clinician awareness of uncomplicated cases of ileal diverticulitis such as this patient presentation will result in improved outcomes for a multitude of future patients.

## Introduction

In Western countries, diverticula often result from the protrusion of the mucosal and submucosal layers of the small and large bowel but are a rarer occurrence in the small intestine, specifically within the ileum [1]. Based on one retrospective review of 208 patients, within the small bowel, the most common regions for diverticula are the duodenum (79%), followed by the jejunum and ileum (13%) [2]. Jejunal diverticula tend to occur more commonly than ileal diverticula with a ratio of 5:1 [3]. Although these diverticula can be asymptomatic and found incidentally on imaging [4], and despite their reduced incidence, jejunoileal diverticula are more likely to be associated with complications such as diverticulitis resulting in perforation and abscess formation [2]. However, jejunal and ileal diverticulitis often present vaguely and differently among patients [5] with varying levels of severity making diagnosis difficult. Due to this, small intestine diverticulitis, especially ileal diverticulitis, is not generally on the differential diagnoses of the acute abdomen and is often mistaken for other more common causes given the standard clinical presentation of fever, abdominal pain, and elevated white blood cell count seen in more prevalent pathologies such as appendicitis [6]. Thus, the diagnosis of ileal diverticulitis often depends on the clinician’s awareness of the disease and the presentation severity [5].

Due to the lower occurrence of ileal diverticula compared to duodenal, jejunal, or colonic diverticula, ileal diverticulitis is an extremely rare disease, with most reported presentations involving complications such as perforation, abscess formation, and fistulas that often require surgical management [6,7]. In contrast to these more complicated presentations, this report illustrates an uncomplicated case of ileal diverticulitis treated successfully conservatively with medical management.

## Case presentation

A 69-year-old female with a surgical history of bilateral tubal ligation and a medical history significant for hypertension, hypercholesteremia, and mild intermittent asthma presented to an outpatient clinic with right lower quadrant (RLQ) abdominal pain for one week and one episode of vomiting. The patient had chills, diaphoresis, nausea, and vomiting preceding the development of waxing and waning sharp RLQ abdominal pain radiating to the right upper quadrant (RUQ) and midline exacerbated by eating. Two days following this, the patient had one episode of non-bloody watery diarrhea. The nausea, vomiting, and diarrhea resolved but the RLQ abdominal pain continued prompting outpatient evaluation. She denied experiencing similar symptoms in the past.

**Figure 1:**
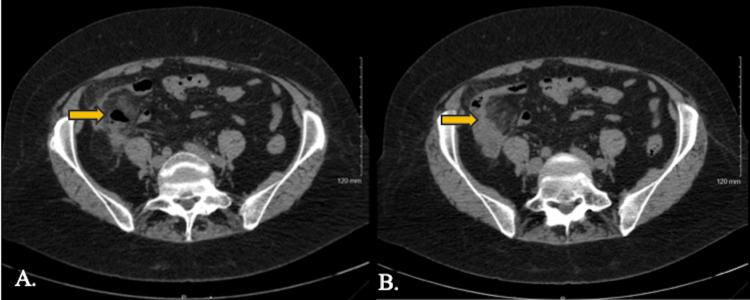
Axial CT scan of the abdomen and pelvis five days after the patient’s presentation in the office. Image A is cephalad to image B. The arrow in A depicts diverticular outpouching in the distal ileum. The arrow in B indicates amorphous fat stranding surrounding the distal ileal loop.

On physical examination, the patient was overweight (body mass index of 25.8 kg/m^2^), afebrile, tachycardiac, and in no distress. She had RUQ and RLQ abdominal tenderness. Her labs were notable for a white blood cell count of 12.6 with increased neutrophils of 10,357 cells/µL and monocytes of 1,071 cells/µL, hemoglobin of 11.5 g/dL, hematocrit of 33.4%, lipase of 77 U/L, and sodium of 132 mmol/L. Other obtained labs were within normal limits including the remainder of complete blood count and comprehensive metabolic panel and amylase. CT of the abdomen/pelvis without contrast was ordered given a differential diagnosis including acute appendicitis but was delayed due to the necessity for prior insurance authorization and was obtained five days after the initial presentation. CT of the abdomen/pelvis demonstrated distal ileal diverticulitis (arrows; Figures 1A, 1B) without evidence of perforation or appendicitis.

Given the patient’s stable clinical condition, a therapeutic intervention was initiated following a return of labs one day after the initial presentation and consisted of ondansetron 4 mg every six hours for repeated episodes of nausea and conservative management with a restricted diet and oral antibiotics including ciprofloxacin 500 mg every 12 hours and metronidazole 500 mg every eight hours. The patient was seen in the office one day following the CT scan, six days after the initial presentation, for reevaluation of symptoms. The patient stated that her symptoms including vomiting, diarrhea, and RUQ and RLQ abdominal pain had completely resolved. She still admitted to a decreased appetite so was instructed to continue with a restricted diet with a gradual reintroduction of bland foods. Repeat physical examination demonstrated vital signs within normal limits and no abdominal tenderness.

## Discussion

In comparison to small bowel diverticulosis, in Western countries, colonic diverticulosis and diverticulitis predominately affect the sigmoid colon [1]. The prevalence of colonic diverticulosis in the United States by age 60 is 60% [1], whereas the prevalence of small bowel diverticulosis within the population is 0.5-2.3% [8]. Both small intestine and colonic diverticula are associated with complications, including diverticulitis, hemorrhage, perforation, abscess formation, and obstruction, although clinical presentation varies, [1,3,4], and, as in this case presentation, may mimic other pathologies. Diverticulitis whether occurring in the small or large intestine often presents similarly with abdominal pain and tenderness, fever, and elevated white blood cell count [6]. Even though small intestine diverticulosis is relatively rare given its comparatively minute prevalence to colonic diverticulosis, resulting pathologies including diverticulitis are still clinically relevant in both an inpatient and outpatient setting often presenting with varying levels of severity.

As a result of the reduced prevalence of small bowel diverticulosis and widespread location of the small intestine in the abdomen on palpatory examination, these presentations are often mistaken for other more common and/or emergent pathologies depending on the region of the symptomatic small bowel diverticulitis. Specifically, terminal ileum diverticulitis resulting in RLQ abdominal tenderness has been mistaken for acute appendicitis, Crohn’s disease, incarcerated inguinal hernias, and other inflammatory conditions that localize to the RLQ [3]. In one case presentation, a patient was taken to the operating room for a suspected right-sided incarcerated inguinal hernia and on exploration was found to have terminal ileal diverticulitis resulting in the resection of the diseased bowel with primary anastomosis [3]. In another case presentation, the patient presented with symptoms concerning for Crohn’s disease including RLQ abdominal pain, leukocytosis, and elevated C-reactive protein with thickening of the distal ileum on CT scan, but upon clinical resolution of symptoms and follow-up MRI, it was determined the patient had ileal diverticulitis [9]. In this case report, the patient’s presentation mimicked mild atypical appendicitis and was thus managed with this diagnosis in mind without the need for more extensive treatment. Ultimately, the initial clinical presentations of all these patients, including the patient in this case, were consistent with more prevalent disease processes; however, the end diagnoses were determined to be due to the same underlying pathology.

Ileal diverticulitis, whether complicated or uncomplicated, should be included in the differential diagnosis of abdominal pain regardless of severity. There have been many previous case presentations regarding the need for greater awareness of complicated ileal diverticulitis in the setting of the acute abdomen necessitating surgical management [5-7,10]. However, as this patient case demonstrates, uncomplicated ileal diverticulitis should not be overlooked and should be included in the differential diagnosis for RLQ abdominal pain as well to prevent or postpone complications. This lack of current awareness in part may be due to the less volatile presentation associated with uncomplicated ileal diverticulitis and thus delayed patient presentation, as was seen with this case. Therefore, increased awareness of this disease process may lead to alterations in patient care management with a decreased need for surgical management as well as improved patient outcomes with a reduction in the current mortality rate (up to 24% in 2020) associated with jejunoileal diverticulitis [7].

In more severe case presentations of ileal diverticulitis complicated by abscesses, perforations, or fistulas, patients underwent surgical management [3,6,7] as this is the current standard of care [4]. There are currently fewer case reports presented that display a patient with uncomplicated ileal diverticulitis and the difference in the management of these patients as compared to more severe cases. In patients with uncomplicated acute small intestine diverticulitis, a restricted liquid diet and selective antibiotics are recommended similarly to colonic diverticulitis in patients at risk for poorer outcomes and those with systemic disturbances or medical comorbidities [4,11]. Yet, due to the varying severity of patient presentations, therapeutic management of patients with ileal diverticulitis is often individualized and can be complicated.

## Conclusions

Although rare, uncomplicated ileal diverticulitis is clinically relevant and should be included in the differential diagnosis of abdominal pain as this disease presentation can cause symptoms that are more commonly associated with more prevalent pathologies such as acute appendicitis. As a result of this mimicry, physicians should have a heightened awareness of this disease process to avoid delayed management or prevent/postpone complications. Overall, although most case reports discuss complicated cases of ileal diverticulitis and their presentation and management, it is still important for clinicians to consider less severe, uncomplicated cases of ileal diverticulitis in the differential diagnosis of abdominal pain. As illustrated by this patient presentation, uncomplicated cases of ileal diverticulitis can be successfully treated conservatively with medical management similar to cases of uncomplicated colonic diverticulitis. However, it is important to note that as with any patient’s care, proper treatment must be individualized, especially given the variable nature of patient presentations with ileal diverticulitis. All in all, one hope is that improving clinician awareness of uncomplicated cases of ileal diverticulitis such as this patient presentation will result in improved outcomes for a multitude of future patients.
